# Comparative Study on the Combustion Behavior and Mechanisms of Ti150 and TC11 Alloys in Oxygen-Enriched Environments

**DOI:** 10.3390/ma18194446

**Published:** 2025-09-23

**Authors:** Xiaohui Zha, Kaikai Feng, Yang Wang, Yuchen Yang, Xin-Yun Zeng, Cheng Zhang

**Affiliations:** 1 AECC Hunan Aviation Powerplant Research Institute, Zhuzhou 412002, China; zhaxh19900605@163.com (X.Z.); fengkai19910207@163.com (K.F.); 18073366886@163.com (Y.Y.); zxy19972025@163.com (X.-Y.Z.); 2 State Key Laboratory for Advanced Metals and Materials, University of Science and Technology Beijing, Beijing 100083, China; m202321341@xs.ustb.edu.cn

**Keywords:** high-temperature titanium alloy, oxygen-enriched combustion, flame propagation mechanism, element enrichment

## Abstract

Ti150 has potential applications in aeroengine components. However, the lack of research on its flame resistance, combustion behavior, and mechanisms makes it difficult to assess the risk of “titanium fire” and leaves fire protection design without theoretical support. This study aimed to determine the combustion resistance of Ti150 and elucidate its combustion behavior and mechanisms to address these issues. Through comparative Promoted Ignition-Combustion (PIC) tests between Ti150 and TC11 alloys, microstructural characterization, and thermodynamic/kinetic analyses, the following conclusions were drawn. Ti150 alloy exhibited a higher critical oxygen pressure and a higher ignition temperature but a significantly faster burning velocity, compared with TC11 alloy. The relationship between pressure and ignition temperature was in good agreement with the modified Frank-Kamenetskii ignition model. The ignition activation energy of Ti150 alloy was determined to be 118.41 kJ/mol, which was approximately 21% higher than that of TC11 alloy (97.72 kJ/mol). Moreover, post-combustion microstructural observations of Ti150 alloy revealed a higher oxygen content in the melting zone and an enrichment of Zr at the solid–liquid interface, both of which contribute to the higher burning velocity of Ti150 alloy compared with TC11 alloy.

## 1. Introduction

Titanium alloys are critical materials for aeroengines and are extensively utilized in core components, such as blades and compressor disks, due to their low density, high strength, and exceptional high-temperature performance [1,2]. However, due to the intrinsic high sensitivity to oxygen of titanium, these titanium-made components, such as internal blades and high-pressure compressor disks, face a risk of combustion as they are exposed to extreme conditions such as high temperature and pressure, high-speed friction or rubbing [3]. These extreme conditions often originate from unexpected situations such as abnormal vibration, failure, or even fracture in the blade-casing system, and can induce catastrophic combustion of titanium alloys, known as “titanium fire” accidents, which seriously threaten the safety and reliability of aeroengines [4,5,6]. Consequently, the combustion behavior of titanium alloys under complex operating conditions is a core issue that urgently needs to be addressed.

Previous research focused on the combustion behavior and mechanisms of commonly used titanium alloys, such as TC4, TC11, TC17, and TC25G. Wang B et al. [7,8] observed that during combustion, titanium alloys such as TC4 and TC11 typically emitted a dazzling white light and numerous sparks while experiencing a sharp temperature increase. The combustion then stabilized into a state characterized by a continuously dripping molten pool, with the dripping rate increasing as oxygen pressure rose. Based on these phenomena, researchers categorized the combustion process into four stages: oxidation, ignition, stable combustion, and extinction [9,10]. In previous studies, the combustion behavior and mechanisms of TC4, TC11, TC17, TC25G, and Ti14 were investigated in detail. Wang [11] conducted a comprehensive comparative study of pure Ti, TC4, TC11, and TC17. The investigation indicated that the ignition temperature among these alloys follows the sequence: TC17 > TC11 > TC4 > Ti, and the burning velocity follows the sequence: TC17 > Ti > TC4 > TC11. The combustion mechanism of titanium alloys is highly complex, depending on multiple parameters such as pressure, oxygen concentration, geometry, dimensions, composition, and phase constitution. Semenov’s thermal ignition theory was successfully applied to describing the relationship between the oxide layer thickness and the ignition temperature during the ignition of titanium particles [12], as well as describing the influence of parameters such as contact radius and flame retardant layer thickness on ignition during rotor-stator friction processes [13]. Some studies attempted to further apply Semenov’s thermal ignition theory to bulk titanium alloys. However, as the ignition criterion ignored the heat transfer within bulk materials, it led to deviations between the predicted critical ignition temperatures and experimental data [14]. Recently, a modified ignition model considering multiple factors, such as sample size, pressure, oxygen concentration, and grain boundaries, was developed based on the Frank-Kamenetskii thermal explosion theory [15,16,17]. This model was applied to describing the ignition conditions of many commonly used titanium alloys [18,19]. Moreover, regarding combustion kinetics, Shao et al. [20,21] investigated the combustion behavior of TC4 and TC11 alloys. They proposed that variations in oxygen concentration within the melting zone alter the peritectic reaction mechanism, thereby affecting the exothermic behavior of the reaction. Simultaneously, different alloying elements, such as vanadium (V) in TC4 and molybdenum (Mo) in TC11, segregate at the solid–liquid interface, which increases the melting point of the interface and reduces the burning velocity. Furthermore, Jain [22] found that vanadium can also form volatile V_2_O_5_, the evaporation of which removes substantial heat and may consequently inhibit the combustion process to some extent [23].

Recently, with the increase in efficiency of aeroengines, the service temperature of high-pressure blades and casings has gradually increased up to 873 K. In order to increase the service temperature of titanium alloys, Ti150 alloy has been developed. It exhibits excellent high-temperature strength, creep resistance, and thermal stability, and is suitable for manufacturing stators and rotors of aeroengines under high-temperature service conditions [24,25]. It thus possesses application potential in the material selection for aeroengine components. Currently, research on Ti150 high-temperature titanium alloys mainly focuses on three areas: microstructure control, heat treatment processes, and mechanical property improvement [26,27,28,29], while research on its combustion resistance and combustion mechanisms remains relatively scarce. However, “titanium fire” has long posed a threat to the service safety and reliability of aeroengines; this research gap introduces significant uncertainties and potential risks to the practical application of Ti150 alloy. and also results in a lack of theoretical basis for titanium fire prevention design. Therefore, a comparative study on the combustion behaviors and mechanisms of Ti150 alloy and commonly used dual-phase titanium alloys (e.g., TC11) is vital for the safe use of Ti150 alloy.

To address the above issues, this study focused on investigating the combustion behaviors (including ignition conditions and combustion rate) and combustion mechanisms (including ignition thermodynamics and combustion propagation kinetics) of Ti150 alloy, and selected the commonly used TC11 alloy as the control group. This study designed combustion experiments for Ti150 and TC11 alloys under oxygen-rich conditions to obtain the critical oxygen pressure for samples of different diameters, as well as the ignition temperature and combustion rate under different oxygen pressures. Additionally, this study characterized and analyzed the microstructure and composition of the samples after combustion and discussed the ignition thermodynamics model and the combustion kinetics model.

## 2. Materials and Methods

### 2.1. Experimental Materials

The Ti150 and TC11 alloys used in this study were produced via vacuum melting, hot rolling, and annealing treatment; their chemical composition is listed in Table 1. Rod samples with diameters of 1, 3.2, 5, 8, 10, and 12 mm and a length of 70 mm were machined from Ti150 and TC11 alloy ingots using wire cutting, milling, and grinding.

### 2.2. Promoted Ignition-Combustion (PIC) Tests

**Figure 1:**
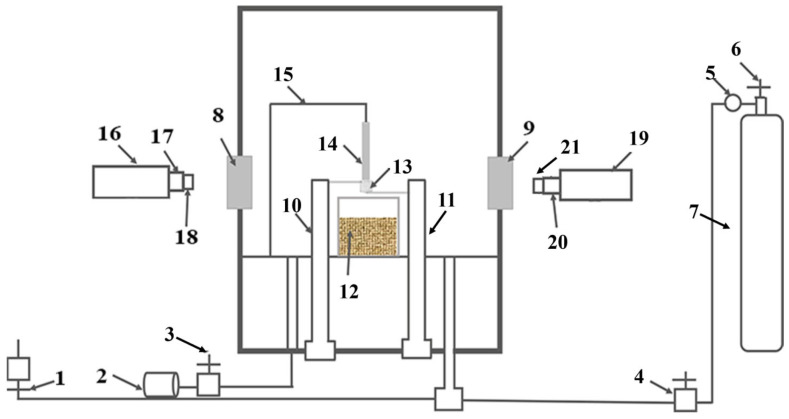
Promoting ignition device schematic diagram: (1) Vent valve; (2) Vacuum pump; (3) Vacuum valve; (4) Inlet valve; (5) Gas pressure reducing valve; (6) Cylinder valve; (7) Oxygen cylinder; (8–9) Observation window; (10–11) Pressure regulator; (12) Quartz crucible; (13) Copper wire; (14) Sample; (15) Bracket; (16) High-speed camera; (17,20) Focus knob; (18,21) Lens; (19) Infrared thermal camera.

This experiment employed a combustion test on Ti150 samples with a PIC-ablation simulation pressure cooker (SP900), which was designed according to the ASTM G-124 standard [30]. A schematic diagram of the apparatus is shown in Figure 1. During the test, a 1 mm diameter copper wire was evenly wrapped around one end of the Ti150 samples and fixed between the two electrodes of the PIC apparatus. After sealing the apparatus, the researchers evacuated the reaction chamber to 10^−2^ Pa. Thereafter, oxygen was introduced into the reaction chamber to reach a predetermined pressure, thereby creating an oxygen-enriched environment. The samples were subsequently ignited through the application of heat to the copper wire using the electrodes. The experiment was repeated by varying the oxygen pressure, and the maximum oxygen pressure at which the samples failed to ignite five consecutive times was defined as the critical oxygen pressure of the samples under oxygen-enriched conditions. Concurrently, temperature changes were recorded through the reaction chamber’s observation port using an Infrared thermal camera (MCS640, Lumasense Technologies, Santa Clara, CA, USA). The ignition temperature of the samples was defined as the point of abrupt temperature increase on the recorded curve. Prior to the tests, the emissivity of the titanium alloy was set to 0.8 via calibration with a dual-color temperature sensor. During the burning velocity tests, a high-temperature-resistant quartz tube was fixed at a point 40 mm from the bottom of the samples to halt combustion. A high-speed camera (PCO.DIMAX S4, PCO AG, Kelheim, Germany) was utilized for recording. The high-speed camera imagery facilitated observation of the detailed process from the ignition of the samples to combustion termination. The combustion reaction time of the samples was recorded, and consequently, the burning velocity of the samples under different oxygen pressures was calculated. The experiment was repeated three times, and the mean burning velocity was calculated to minimize errors.

### 2.3. Microstructure Characterization

The combustion of titanium alloy is characterized by a highly exothermic nature, and once initiated, the combustion persists until the sample is completely consumed. Consequently, argon gas was expeditiously introduced into the combustion chamber during the combustion process to forcibly terminate it, thereby allowing the microstructure of the sample at different stages of combustion to be obtained. Subsequent to the cooling and removal of the sample, the sample’s combustion end was bisected along the longitudinal axis. The resultant sample cross-section was then embedded in epoxy resin and progressively ground and polished with 240–3000 grit sandpapers, 2.5 μm and 0.5 μm diamond pastes, and 0.04 μm colloidal silica suspension. The sample was subsequently etched with an etchant of the precise volumetric ratio of 1:3:6 (HF:HNO_3_:H_2_O). The microstructure and local chemical composition of the post-combustion sample cross-section were characterized using a V (LSCM, VK-X200, Keyence K.K., Osaka, Japan), a field-emission scanning electron microscope (FESEM, SUPRA 55, Zeiss GmbH, Oberkochen, Germany) equipped with an energy-dispersive X-ray spectroscopy (EDS, Aztec X-Max 80, Oxford Instruments PLC, Abingdon, UK) system, and an electron probe microanalyzer (EPMA, 1720H, Shimadzu K.K., Kyoto, Japan). Prior to characterization, the sample was cold-mounted in epoxy resin, affixed to the sample stage using carbon double-sided conductive tape, and sputter-coated with gold to enhance image quality. The SEM and EPMA were both operated at an accelerating voltage of 15 kV. Microstructural examination of various regions was conducted in back-scattered electron imaging (BEI) mode. Raw data were corrected using the XPP method (exponential model of Pouchou and Pichoir matrix correction) in the Aztec 6.1 software for quantitative elemental analysis.

## 3. Results

### 3.1. Combustion Behavior

**Figure 2:**
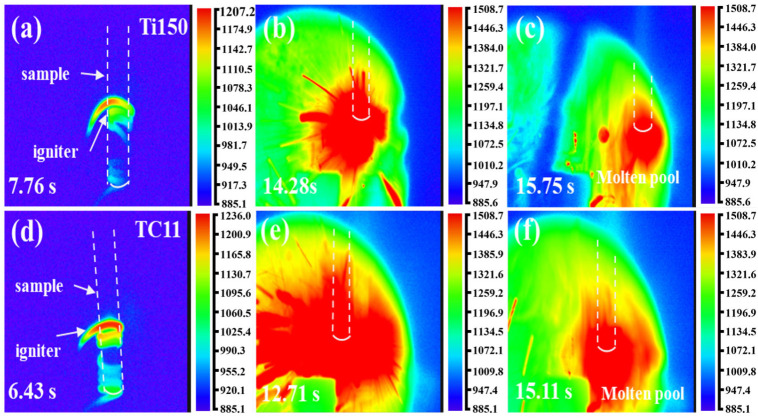
Thermal imaging (in K) of titanium alloy combustion: (**a**–**c**) Ti150 titanium alloy; (**d**–**f**) TC11 alloy.

Figure 2 illustrates the thermal imaging changes in the Ti150 and TC11 titanium alloys during the combustion processes at 0.45 MPa. The images (a–c) depict the Ti150 alloy, while (d–f) illustrate the TC11 alloy; both titanium alloys exhibit similar combustion behavior. The combustion processes of both titanium alloys can be categorized into four stages: thermal oxidation, ignition, flame propagation, and quenching. The thermal oxidation stage (Figure 2a,d) occurs through the delivery of heat to the sample ends by resistance wires, thereby causing the sample to gradually heat up. The ignition stage, as demonstrated in Figure 2b,e, occurs when the internal energy of the sample accumulates to the critical condition, thereby inducing the combustion of the sample. This phenomenon is characterized by the rapid temperature surge, the emission of a bright white light, and the production of droplets and a large amount of smoke. The flame propagation stage is illustrated in Figure 2c,f. Given the high combustion heat of titanium alloys, once ignition occurs, the heat released by the combustion reaction sustains the sample’s continued combustion, with the flame propagating upward until the entire sample is consumed and the flame is extinguished.

**Figure 3:**
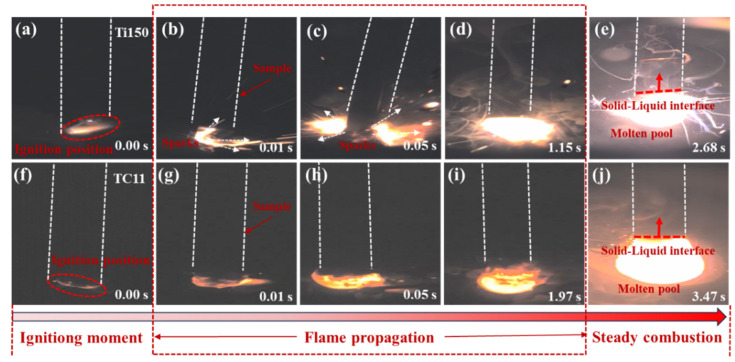
Combustion process diagrams of two titanium alloys: (**a**–**e**) Ti150 alloy; (**f**–**j**) TC11 alloy.

Figure 3a–j illustrates the combustion process of two alloys, from the initial ignition to the formation of a stable molten pool, using a high-speed camera. Figures (a) and (f) indicate that the samples of Ti150 and TC11 did not melt immediately upon ignition. Figures (b–e) and (g–j) show the process of rapid flame expansion after ignition, during which the combustion zone melted into a liquid phase at high temperature, forming a molten pool and achieving stable combustion. At the time of ignition (0.00 s), Ti150 produced larger sparks, while TC11 produced smaller sparks. The time required to reach stable combustion is 2.68 s for Ti150 and 3.47 s for TC11. The Ti150 alloy formed a molten pool more rapidly after ignition; a large amount of droplet splashing and smoke emission occurred during combustion; and it exhibited a higher burning velocity.

**Figure 4:**
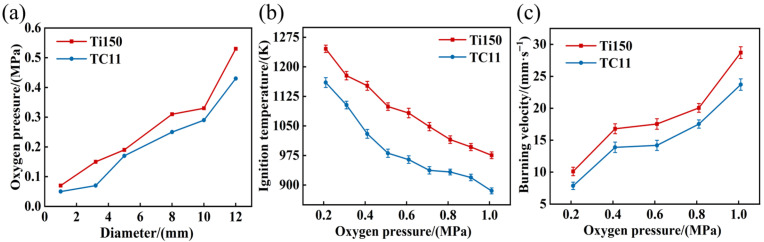
(**a**) Critical oxygen pressure variation curves of samples of different diameters; (**b**) Critical ignition temperature of samples under different oxygen pressures; (**c**) Burning velocities of Ti150 alloy and TC11 alloy under different oxygen pressures.

Furthermore, the critical oxygen pressures and temperatures of Ti150 and TC11 are compared, as illustrated in Figure 4. In Figure 4a, as the sample diameter increases from 1 mm to 12 mm, the critical oxygen pressure of the Ti150 alloy increases from 0.07 MPa to 0.57 MPa, while the critical oxygen pressure of the TC11 alloy increases from 0.05 MPa to 0.43 MPa. At equivalent dimensions, the Ti150 alloy exhibits a higher critical oxygen pressure than the TC11 alloy. Figure 4b illustrates that, as the oxygen pressure increases from 0.21 MPa to 1.01 MPa, the ignition temperature of the Ti150 alloy decreases from 1245.8 K to 975.6 K, while that of the TC11 alloy decreases from 1160.2 K to 885.1 K. Additionally, Figure 4c illustrates the burning velocities of the two materials under different oxygen pressures. It is evident that as the oxygen pressure increases from 0.21 MPa to 1.01 MPa, the burning velocity of the Ti150 alloy increases from 10.11 mm/s to 28.72 mm/s, and that of the TC11 alloy increases from 7.86 mm/s to 23.71 mm/s, showing a marked acceleration in both samples, with the Ti150 alloy burning faster than the TC11 alloy. In summary, compared to the TC11 alloy, the Ti150 alloy exhibits a higher critical oxygen pressure and a higher ignition temperature; once ignited, it burns more vigorously and demonstrates a higher burning velocity.

### 3.2. Microstructural Characteristics After Combustion

**Figure 5:**
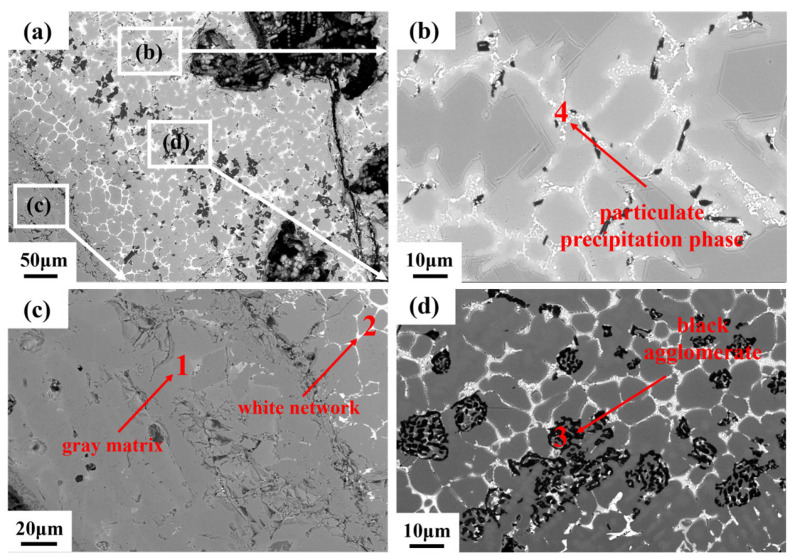
Microstructure of the oxide zone of Ti150, obtained using the BEI mode of SEM: (**a**) overall morphology; (**b**–**d**) partial enlargements of different regions.

The microstructure and local chemical composition of the combusted TC11 alloy are detailed in Reference [20]. After combustion, the microstructure of the combusted Ti150 alloy sample is illustrated in Figure 5. The microstructure can be divided into three regions based on morphology and composition: the oxide zone, the melting zone, and the heat-affected zone. The oxide zone, characterized by numerous pores and cracks, is located at the outermost layer. This zone primarily consists of a gray matrix phase (Phase 1), a white network phase (Phase 2), a black agglomerate phase (Phase 3), and a white particulate precipitation phase (Phase 4). The compositions of these phases are shown in Table 2. The gray matrix phase contains 34.53 at.% Ti and 61.07 at.% O, which corresponds mainly to TiO_2_. The white network phase is distributed around the black agglomerates. According to Table 2, it is enriched in Sn and Nb, with the concentrations of Sn and Nb more than three times higher than those in the gray matrix phase. The black agglomerate phase contains 39.23 at.% Al and 54.60 at.% O, which corresponds primarily to Al_2_O_3_. The white particulate precipitation phase contains high concentrations of O, Zr, and Ti. It possibly consists of zirconium and titanium oxides or their complex oxides.

**Figure 6:**
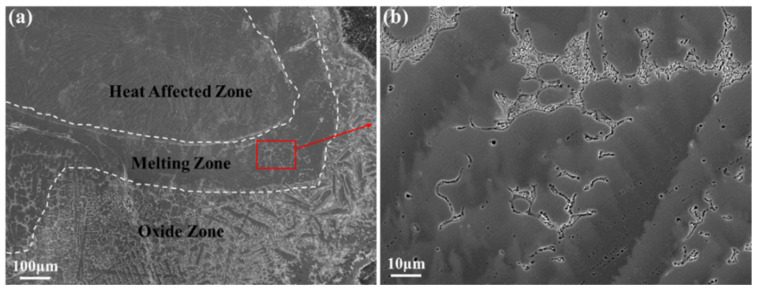
Microstructure of the melting zone of Ti150, obtained using the BEI mode of SEM: (**a**) overall morphology; (**b**) partial enlargements.

**Figure 7:**
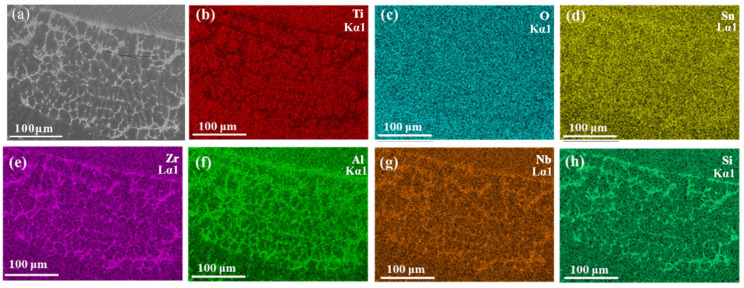
Melting zone of Ti150: (**a**) Microstructure obtained using the BEI mode of SEM; (**b**–**h**) Elemental distribution maps acquired by EDS mapping.

The melting zone of the Ti150 alloy exhibits a typical dendritic microstructure, comprising a dark matrix phase and a bright dendritic phase, as illustrated in Figure 6. The width of the melting zone in the Ti150 alloy is approximately 100–150 μm, whereas the melting zone in the TC11 alloy is only 20–25 μm wide. Figure 7 further shows the EDS elemental mapping in the melting zone. The light dendritic phase is enriched with Al, Zr, Nb, and Si, while the dark matrix phase is enriched with Ti. This is different from that observed in the single phase in the melting zone of the TC11 alloy [20]. Additionally, Table 3 presents the chemical composition for the dark matrix phase in the melting zone of the Ti150 alloy. Its oxygen content reaches 31.43 at.%, compared to 27.43 at.% in the dark matrix phase of the TC11 alloy, indicating a slightly higher oxygen concentration in the melting zone of the Ti150 alloy.

**Figure 8:**
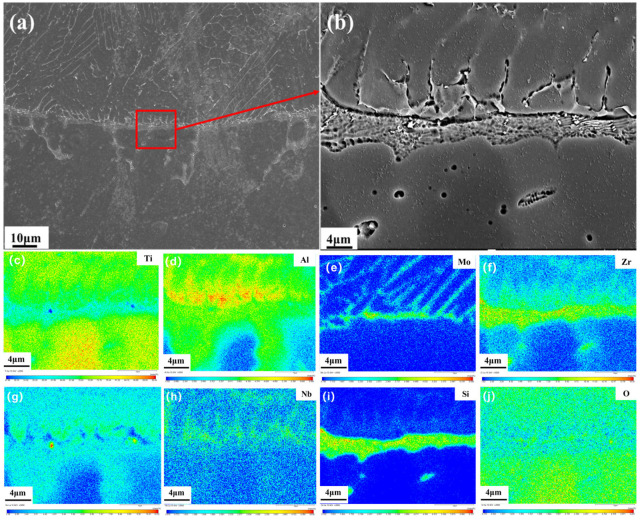
Solid–liquid interface of Ti150: (**a**) overall morphology using the BEI mode of SEM; (**b**) partial enlargements using the BEI mode of SEM; (**c**–**j**) Elemental distribution maps acquired by EPMA mapping.

Figure 8a shows the morphology at the solid–liquid interface of Ti150. As can be seen, the grains in the heat-affected zone grow larger due to heating during combustion. A distinct solid–liquid interface exists between the heat-affected zone and the melting zone. On the side closer to the melting zone is a narrow, gray strip-like structure. On the side closer to the heat-affected zone is a gray-white, network-like structure. EPMA testing was conducted on an enlarged image of the solid–liquid interface to determine element distribution. The results are shown in Figure 8b. The narrow strip-like structure is primarily enriched with Zr and Si, and the grayish-white network-like structure is primarily enriched with Al, Mo, and Sn. The specific composition of the solid–liquid interface is shown in Table 4. The solid–liquid interface of Ti150 exhibits enrichment of 6.43 at.% Zr and 2.43 at.% Mo, whereas that of TC11 exhibits enrichment of 1.39 at.% Zr and 9.3 at.% Mo [20].

## 4. Discussion

### 4.1. Comparison of Thermodynamic Parameters for Ignition of Ti150

In order to determine reaction order and activation energy, the relationships between sample diameter and critical oxygen pressure, as well as between oxygen pressure and ignition temperature, were fitted by using the modified Frank-Kamenetskii ignition model reported in our previous work [19], and its expression is as follows:

(1) $$\delta =\frac{E{l_{m}}^{2}}{\lambda RT^{2}}k{q^{{}^{\circ}}}_{r}\frac{{\left(C_{i}\frac{P}{P_{a}}\right)}^{n}}{1+\alpha {\left(1-C_{i}\right)}^{n}P^{n}}\exp(\frac{-E}{RT})$$

In the equation, *k* is the pre-exponential factor, *E* is the activation energy, *R* is the molar gas constant, *λ* is the thermal conductivity of the sample material, $l_{m}$ is the length of the sample, ${q^{{}^{\circ}}}_{r}$ is the heat generation rate of the sample material during ignition, *n* is the reaction order, *α* is the adsorption coefficient, $C_{i}$ is the oxygen concentration, *P* is the critical oxygen pressure, and $P_{a}$ is the atmospheric pressure. According to formula (1), the critical ignition condition is affected by the sample diameter and the ambient oxygen pressure. The relationship between sample diameter and critical oxygen pressure can be expressed as:

(2) $$\ln\frac{P}{P_{a}}=\frac{1}{n}\ln\left(a^{2}+A\right)+\frac{1}{n}\ln B$$

In the equation, *a* is the diameter of the sample, $A=\frac{1.68}{0.84}c^{2}$, $B=\frac{E{q^{{}^{\circ}}}_{r}{l_{m}}^{2}}{\lambda RT^{2}}kc^{2}exp\left(-\frac{E}{RT}\right)$, which is usually regarded as a constant. The relationship between oxygen pressure and critical ignition temperature under oxygen-enriched conditions can be expressed as:

(3) $$ln\frac{P}{P_{a}}=\frac{2}{n}lnT+\frac{Y}{T}+Z$$

In the equation, $Y=\frac{E}{RT}$, $Z=\frac{1}{n}ln(\frac{\delta_{c}\lambda R}{E{l_{m}}^{2}k{q^{{}^{\circ}}}_{r}})$ can be regarded as a constant.

**Figure 9:**
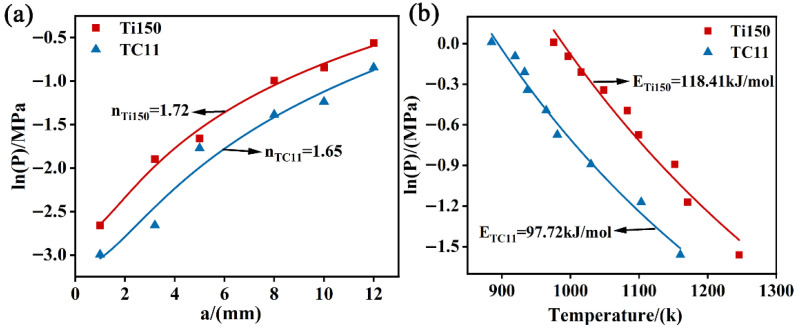
(**a**) Fitting of the relationship between sample diameter and critical oxygen pressure; (**b**) Fitting of the relationship between oxygen pressure and ignition temperature.

The experimental data were fitted using Equations (2) and (3), and the results are shown in Figure 9. The reaction order for the Ti150 alloy is 1.72, with an activation energy of 118.41 kJ/mol; the reaction order for the TC11 alloy is 1.65, with an activation energy of 97.72 kJ/mol. The activation energy of the TC11 alloy is lower than that of the Ti150 alloy, indicating that the Ti150 alloy has lower sensitivity to combustion as compared to the TC11 alloy under the same oxygen-enriched conditions.

### 4.2. Flame Propagation Mechanism of Ti150 Alloy

The Ti150 alloy has lower sensitivity. However, once ignited, it burns faster than the TC11 alloy. The main factors for this phenomenon are: first, the oxygen content in the melting zone, and second, the element distribution at the solid–liquid interface. The burning velocity of titanium alloys is manifested microscopically as the migration rate of the solid–liquid interface. As posited in prior studies [19], the migration rate of the solid–liquid interface can be calculated by the following formula:

(4) $$v=\frac{\bar{q}}{\left\{\rho \left[c\left(T_{m}-T_{0}\right)+\lambda_{m}\right]\right\}}\cdot \frac{\bar{A}}{S}$$

In this equation, $\bar{q}$ represents the heat flowing out of the melt pool, $\bar{A}$ represents the solid–liquid interface area, *ρ* represents the density of the titanium alloy, *c* represents the specific heat capacity, $T_{0}$ represents the initial temperature of the sample, *S* represents the cross-sectional area of the sample (related only to the diameter of the sample), and $T_{m}$ represents the melting point of the solid–liquid interface. The migration rates are denoted as $\upsilon_{1}$ for Ti150 and $v_{2}$ for TC11. To compare the migration rates of the two alloys, a proportionality coefficient *η* is introduced, as shown below:

(5) $$\eta =\frac{\upsilon_{1}}{v_{2}}=\frac{{\bar{q}}_{1}}{{\bar{q}}_{2}}(1-\frac{C\Delta T_{m}}{C\left({T_{m}}_{1}-T_{0}\right)+\lambda_{m}})$$

In this equation, $∆T_{m}={T_{m}}_{1}-T_{m2}$, when $\eta >1,\;\upsilon_{1}>\upsilon_{2}$.

**Figure 10:**
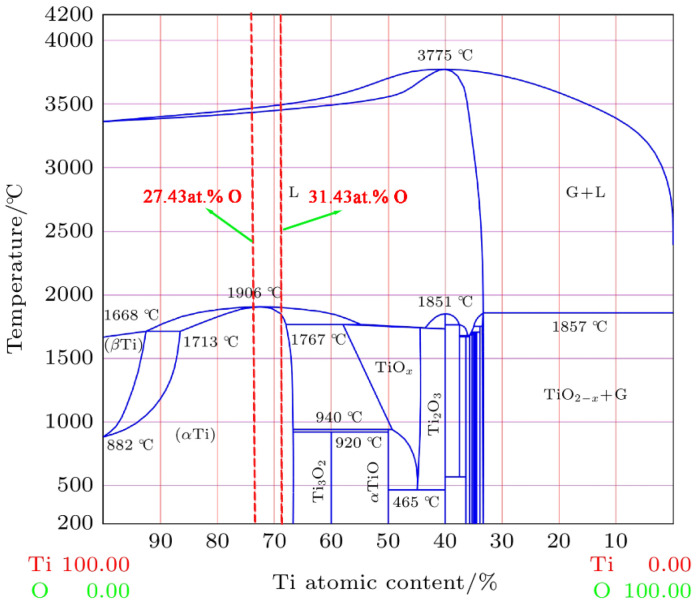
Ti-O equilibrium phase diagram [31].

First, since Ti150 alloy is a near-α-type titanium alloy and TC11 is an α+β-type titanium alloy, the phase contents of the two alloys are different. According to the Ti-O equilibrium phase diagram shown in Figure 10 [31], the maximum solid solubility of oxygen in β-Ti is about 8 at.%, and the maximum solid solubility in α-Ti can reach about 33 at.%. The increase in α phase enhances the solid solubility of oxygen in the samples [32,33,34]. In the melting zone, this effect is manifested as more oxygen entering the Ti150 alloy during the combustion process. The O content in the Ti150 melting zone (31.43 at.%) is slightly higher than that in the TC11 melting zone [20]. Since the heat release of oxides is positively correlated with the oxygen content, the heat transferred through the solid–liquid interface in the Ti150 melting zone is greater than that in the TC11 melting zone, that is, ${\bar{q}}_{1}>{\bar{q}}_{2}$.

Furthermore, the melting temperature at solid–liquid interfaces of two alloys is affected by the enrichment of different elements, resulting in different burning velocities. It can be seen from Figure 8 and Table 4 that the solid–liquid interface of Ti150 alloy is enriched with 6.43 at.% Zr, which is higher than the Zr content of TC11 (1.39 at.%). It can be seen from the Ti-Zr binary phase diagram [35] that at this content, Zr causes the melting point of the solid–liquid interface to drop to about 1890 K. Under the same oxygen pressure, the solid–liquid interface of TC11 alloy is enriched with 9.30 at.% (20.79 wt.%) Mo, which is higher than the Mo content of Ti150 (2.43 at.%), causing the solid–liquid interface temperature to rise to about 1980 K [20]. Therefore, the melting point of the solid–liquid interface of Ti150 is reduced by about 50 K compared to the melting point of pure titanium (≈1940 K) due to the influence of Zr, and the melting point of the solid–liquid interface of TC11 is increased by about 40 K compared to the melting point of pure titanium due to the influence of Mo, making ${T_{m}}_{1}<T_{m2},∆T_{m}<0$.

In summary, the combined influence of heat release differences and alloying elements results in a faster burning velocity for Ti150 compared to that of TC11. Due to its higher α-phase content, the Ti150 alloy allows more O to dissolve into the melting zone, increasing the heat released and accelerating the burning velocity of Ti150. The enrichment of Zr at the solid–liquid interface in Ti150 lowers the melting point there, while the enrichment of Mo at that interface in TC11 raises the melting point. According to Equation (5), η>1, $\upsilon_{1}>\upsilon_{2}$, meaning the Ti150 alloy burns faster than the TC11 alloy, which is consistent with the experimental results.

Overall, the Ti150 alloy demonstrates better combustion resistance compared to the TC11 alloy. However, it burns more quickly once ignited. In practical applications, due to its excellent high-temperature mechanical properties and the performance demands of modern aeroengines, it is exposed to severe working conditions such as higher temperatures, increased rotational speeds, and greater pressures. This study focuses only on the combustion behavior and mechanisms of the Ti150 alloy samples in an oxygen-rich environment. Further research is needed on the ignition risk, burning velocity, and flame propagation under actual service conditions involving multi-field coupling. The material should be used only after its reliability has been confirmed. Where necessary, combustion-resistant coatings should be applied for protection to reduce the risk of ignition and prevent consequences caused by fire propagation.

## 5. Conclusions

This study employed the PIC test to compare the combustion resistance of Ti150 with that of TC11 by recording their combustion behaviors (including combustion images, ignition conditions, and combustion velocities). Furthermore, based on the ignition thermodynamics and the combustion kinetics models, this study comparatively analyzed the combustion mechanisms of Ti150 and TC11. The main conclusions are as follows:

1. The ignition phenomenon of the Ti150 alloy is analogous to that of typical titanium alloys. The critical ignition pressure and ignition temperature of Ti150 are both higher than those of the TC11 alloy. As the oxygen pressure increases, the burning velocity of the Ti150 alloy gradually increases. At the same oxygen pressure, the burning velocity of the Ti150 alloy is higher than that of the TC11 alloy.
2. The Ti150 alloy exhibits significant differences from TC11 in terms of both the characteristics of its melting zone and the elemental enrichment at its solid–liquid interface. The melting zone of Ti150 is considerably wider (100–150 μm). Furthermore, at the solid–liquid interface, marked Zr enrichment is observed.
3. The thermodynamic parameters of Ti150 and TC11 were obtained based on the ignition thermodynamic model. The ignition activation energy of the Ti150 alloy (118.41 kJ/mol) exceeds that of the TC11 alloy (97.72 kJ/mol), suggesting that the Ti150 alloy exhibits better combustion resistance. The higher burning velocity of Ti150 compared to TC11 is mainly attributed to two factors: firstly, the Ti150 alloy has a higher O content in the melting zone, which leads to increased reaction exotherm; secondly, the enrichment of Zr at the solid–liquid interface in Ti150 leads to a lower melting point. In contrast, Mo in TC11 exerts the opposite effect. The combined effect of these factors accelerates the burning velocity of Ti150 relative to TC11.

Solely from the perspective of ignition risk, replacing TC11 with Ti150 does not increase the risk of “titanium fire.” However, as future aeroengines’ operating temperatures rise and tip clearances decrease, the necessity for titanium fire prevention measures will continue to increase. The combustion mechanism elucidated in this paper may still provide a partial reference for such research. However, due to the extremely harsh service conditions of high-pressure compressors and the high cost of experimental replication, this study necessarily simplified the samples’ geometric characteristics and the test parameters. Future work should focus on refining experimental methods, incorporating additional parameters (e.g., heating conditions, power, and gas flow rate), improving thermodynamic and kinetic models, and establishing correlations between laboratory samples, simulated parts, and actual engine components. Such efforts are essential to mitigating combustion risks when applying new titanium alloys in aeroengines.

## Figures and Tables

**Table 1:** Chemical compositions of Ti150 and TC11 alloys.

Material	Composition (wt%)					
	Al	Sn	Zr	Mo	Nb	Si
Ti150	5.8	4	3.4	0.5	0.7	0.35
TC11	6.5	0	1.5	3.5	0	0.3

**Table 2:** Chemical composition of different phases in the oxide region.

Region	Composition (at%)							
	Ti	Al	Sn	Zr	Mo	Nb	Si	O
Phase 1	34.53	2.56	0.03	1.64	0.04	0.13	0	61.07
Phase 2	53.11	5.06	7.23	2.43	0.92	1.07	3.41	26.76
Phase 3	4.49	39.23	0.09	1.42	0.03	0.06	0.08	54.6
Phase 4	18.27	4.77	1.23	23.76	0.22	0.34	0.24	51.16

**Table 3:** Chemical composition of the melting zone of Ti150.

Melting Zone	Composition							
	Ti	Al	Sn	Zr	Mo	Nb	Si	O
wt%	70.51	4.30	3.54	6.78	0.57	1.12	0.26	12.93
at%	57.27	6.20	1.16	2.89	0.23	0.47	0.36	31.43

**Table 4:** Chemical composition of the solid–liquid interface.

Solid–Liquid Interface	Composition							
	Ti	Al	Sn	Zr	Mo	Nb	Si	O
wt%	66.3	5.8	2.9	12.2	4.9	2.5	3.5	1.9
at%	66.6	10.34	1.17	6.43	2.43	1.29	5.99	5.71

## Data Availability

The original contributions presented in this study are included in the article. Further inquiries can be directed to the corresponding author.
